# Narrative Elicitation as Ethnography: Methodological Insights From the Examination of Children's Perspective Marking in Amdo Tibetan

**DOI:** 10.3389/fpsyg.2021.644331

**Published:** 2021-06-03

**Authors:** Shannon M. Ward

**Affiliations:** Department of Community, Culture, and Global Studies, University of British Columbia Okanagan, Kelowna, BC, Canada

**Keywords:** language socialization, ethnography, narrative elicitation, language endangerment, perspective marking, evidentiality, Tibetan language

## Abstract

This paper employs a case study with Amdo Tibetan children to demonstrate the benefits of narrative elicitation for ethnographic language socialization research in under-studied languages. Primarily by examining spontaneous verbal interaction, existing language socialization research has demonstrated how salient grammatical resources shape children's understanding of cultural belief systems pertaining to sociality and the appropriate display of emotion. However, spontaneous data do not always capture children's full linguistic repertoires and competencies, and may therefore present a partial picture of their mastery over particular grammatical systems. One such area that remains to be studied is how children use interactional cues to build their emerging knowledge of grammatical perspective marking in Tibetan languages. This paper integrates narrative elicitation with ethnographic methods from language socialization to examine how Amdo Tibetan children mark perspective using evidentiality, the grammatically-obligatory encoding of knowledge source, an area not frequently documented in language socialization studies. Language socialization research involved 15-months of participant observation, audio-video recording, and analysis of spontaneous interactions with children aged 1–4. This ethnographic research found that adults' narratives highlighted local theories about the importance of compassion (Tib. *snying rje*) by using grammatical evidentiality to emphasize characters' direct experiences in the story-world. However, grammatical evidentiality was under-represented in children's spontaneous talk. To provide further insight into children's mastery of evidentiality in this culturally salient communicative genre, I conducted narrative elicitation tasks with seven Amdo Tibetan children, aged 2–7. By framing narrative elicitation tasks as forums for social interaction in family homes, I adapted a method traditionally used in experimentation to complement the study of naturalistic interaction. Interaction analysis of the elicited narratives found that family members positioned young children as novice narrators, leading to dialogic rather than monologic narratives. Young children co-constructed shared perspectives on narrated events, and used evidentiality in conventionalized ways by mirroring the grammatical forms of adults' previous utterances. By adapting narrative elicitation tasks to language socialization's ethnographic methods, this paper models how qualitative researchers can locate patterns in children's experiences of language across complementary settings of data collection, an endeavor that is particularly important to research with child speakers of under-documented languages.

## Introduction

This paper employs a case study from an Amdo Tibetan community (Qinghai, China) to demonstrate how narrative elicitation can be used in the ethnographic investigation of young children's language development. The paradigm of language socialization provides us with the most widely accepted, comprehensive methodology for conducting ethnographic research on language acquisition, especially (but not exclusively) on young children's first language acquisition (Schieffelin and Ochs, 1984). Language socialization researchers share the conviction that language systems emerge through social interaction. As a result, children's experiences of language are inseparable from the everyday communicative routines and belief systems that constitute the cultures they are raised in. Grounded in the discipline of Anthropology, language socialization researchers employ specialized ethnographic methods involving four key features: (1) longitudinal research design; (2) field-based collection and analysis of audio-visual recordings; (3) sociohistorical contextualization of data; and (4) consideration of the links between immediate settings of interaction, and social and political systems (Garrett, 2008). While the first two features show significant methodological overlap with field linguistics and language documentation, the second two features are shared by cultural and linguistic anthropology more broadly.

Language socialization's ethnographic attention to local communicative practices lends itself to a preference against working with elicited data (Miller et al., 2014, p. 190). The explicit focus on spontaneous verbal interaction has led to major advances in our understandings of the dialectical relationship between language and cultural systems. At the same time, language socialization's expansive theoretical orientation leaves room for additional modes of inquiry. In interpreting spontaneous language data, language socialization scholars draw together interdisciplinary theories, including developmental psychology's attention to attachment and relationality in the socialization of emotion (Clancy, 1986; Fung, 1999; Takada, 2019), educational psychology's interest in the interactional construction of identity (Cook, 1996; He, 2004), and cultural psychology's incorporation of experimental tasks into participant observation (Rogoff et al., 1998; Xu, 2019). Language socialization research with naturalistic data has documented how local communicative routines shape young children's emotional experiences and social relationships, a point of interest that unites the disciplines of Psychology, Education, and Anthropology.

However, spontaneous data do not always demonstrate children's full linguistic competencies, and may therefore present a partial picture of children's mastery over key grammatical systems. One such area that remains to be studied holistically is how young children use interactional cues in grammatical perspective marking. Children (and other language learners) only begin to produce syntactic forms well after they comprehend them (Clark, 2009, p. 14). Therefore, young children may not readily use all grammatical resources that are salient in the language input they encounter. In communities where languages are shifting and we have limited documentation of key grammatical resources, it is particularly pressing to uncover children's full knowledge of how to mark perspective and display emotion in line with cultural belief systems.

Existing literature has addressed socialization into community expectations surrounding perspective-marking and emotional expression by examining content-based and grammatical patterns in adults' speech to children, as well as children's multimodal contributions to spontaneous narratives. For example, Burdelski and Mitsuhashi (2010), Clancy (1999), and Suzuki (1999) addressed how Japanese teachers and caregivers use grammatical resources to shape children's emotional alignment with evaluations. Lo (2009) showed how, in a Korean heritage language school, teachers used grammatical evidentiality to evaluate students as morally worthy or suspect. Studies of American (Hérot, 2002), Chinese (Huang, 2011), and Japanese (Takada, 2019) family interactions found that caregivers use affect lexicon, or vocabulary related to emotion, to guide children's behavior and provoke their expressions of empathy. Everyday narrative is a particularly rich discourse genre for enacting emotion socialization. When narrators report and re-enact their own or others' responses to past events, they provide a model for expected emotional reactions (Ochs and Capps, 2001, p. 135–155). For example, Miller's et al. (1996) comparison of everyday narratives in American and Taiwanese families shows how caregivers tell stories *about* children that portray others' emotional reactions to their past actions. Morita (2019) demonstrated that even very young toddlers actively respond to such everyday storytelling, using movement and the physical environment to claim co-tellership and assert their knowledge when adults are speaking about them. These studies demonstrate that, as children build communicative competence, they learn to associate specific grammatical resources with emotional displays. Also, when children learn to use grammatical resources to express emotions in culturally specific ways, they internalize systems of moral value (Clancy, 1986; Lo and Fung, 2014).

Although this existing research has addressed the coordination of emotion in light of differences in adults' and children's repertoires, spontaneous data do not always^1^ demonstrate children's full mastery over salient grammatical systems. As Ochs (1986, p. 835) emphasizes, social values and culturally shaped subject positions motivate and constrain language repertoires. Therefore, identifying the values that a community associates with specific grammatical systems and aged subject positions (Berman, 2019) can explain the presence or absence of certain features in child language. Regardless of language input, children may avoid producing grammatical systems or entire codes (Meek, 2008) that are inconsistent with their identities. These cultural effects on repertoire intersect with the accepted crosslinguistic finding that children may produce syntactically embedded features, such as evidentiality, later in their chronological development. Crosslinguistic studies using psycholinguistic methods suggest that children produce direct evidential marking around age two, but do not produce contrastive evidential marking until after age 4 (Aksu-Koç, 1988 on Turkish; Courtney, 2014 on Quechua), and may not comprehend evidential contrasts until middle childhood (de Villiers and Garfield, 2009 on Central Tibetan). When salient socializing routines involve grammatical systems that are under-represented in children's spontaneous repertoires, additional methods are required to clarify children's full communicative competence. As Andersen (2014) found, for example, semi-structured play routines that position participants in a range of social roles can illuminate children's knowledge of how to mark registers that are inconsistent with their aged identities.

In this paper, I build on literatures from language socialization and the pragmatics of evidentiality to suggest a supplementary method for examining children's uses of key grammatical systems. With a case study of perspective marking in Amdo Tibetan narratives, I demonstrate how narrative elicitation tasks can clarify children's socialization to use culturally valued language structures. As recent work in documentary linguistics suggests, adults' collaborative approaches to elicitation tasks can provide insight into a language's encoding of social cognition (San Roque et al., 2012). Using a similar lens, I present narrative elicitation tasks as socially and culturally situated activities, in an ethnographic setting where there is an obvious mismatch between children's and adults' linguistic repertoires. By employing interaction analysis, this paper positions semi-structured narrative elicitation as an ethnographic method, which can be used to locate patterns in children's experiences of language across complementary settings of data collection. By taking local concerns over children's social and linguistic development as a starting point for defining the object of analysis, this paper offers a research design that is community-based. It also provides a new orientation to a method generally considered to be experimental rather than ethnographic. This approach is particularly well-suited to addressing the language experiences of child speakers of under-documented languages, in settings where language shift threatens the intergenerational continuity of communicative routines.

## Materials and Methods

This section describes the data set examined in the results, details the methods used to collect and interpret the data, and outlines the interpretive decisions that motivated the use of semi-structured elicitation.

### The Data Set

The data examined in this paper include a collection of ten elicited Frog Story narratives, told by seven Amdo Tibetan children, aged two through seven, with their caregivers and/or siblings (Table 1). I examined children's Frog Story narratives alongside five folktales elicited from young adults. I contextualized these narrative elicitations with adults' descriptions of the functions of narrative in childrearing. Two of the children were enrolled as focal participants in a related longitudinal research project. The other five child participants and the adult participants were recruited from among these two children's community members.

**Table 1:** Frog story elicitations by primary teller.^1^

**Name**	**Sonam**	**Sakya**	**Lhamo**	**Tashi**	**Dawa**	**Dolma**	**Yeshi**
Age at elicitation	2;8	4;0	4;2, 4;5, and 5;7	4;4	5;10	5;10 and 7;3	6;9
Date(s) of elicitation	6/11/2017	3/18/2017	1/22/2017; 4/30/2017; 6/18/2018	6/21/2017	1/22/2017	1/22/2017; 6/18/2018	4/23/2017

The narrative elicitation procedure sought to document children's grammatical repertoires in socially-situated experiences of storytelling. The elicitation procedure used a picture book prompt to present one child narrator, the primary teller, with a structured event sequence. I hypothesized that using a picture book prompt would encourage children to produce evidentiality, which was salient in adult narratives to children, but under-represented in children's spontaneous talk.^2^ Evidentiality refers to the grammatically-obligatory marking of knowledge source. Evidentiality involves perspective marking, and is therefore a potent resource for articulating emotional displays. As elaborated below, the Amdo Tibetan evidential system is integrated with tense/aspect. I hypothesized that, because children's everyday talk tended to feature directives and remain focused on present activities, evidentiality was under-represented in spontaneous data. I hypothesized that a set of temporally sequenced images would prompt children to produce evidentiality. To examine this hypothesis, I piloted a narrative elicitation task with two children who were focal participants in the longitudinal study, Lhamo (age 4;2) and Dolma (age 5;10). In these first narrative elicitations, Lhamo and Dolma marked perspective with evidentials, revealing uses of grammar that were under-represented in their spontaneous talk. Due to the success of these initial samples, I recruited five additional child participants for narrative elicitation.

Mercer Mayer's (1969) wordless picture book *Frog, Where Are You?*, which has been used frequently in crosslinguistic studies (Bamberg, 1987; Stromqvist and Verhoeven, 2004), served as the elicitation prompt. In traditional Frog Story methods, participants are unfamiliar with the picture book before beginning their narratives. Participants are then instructed to look through the entire picture book. After viewing all of the pages, participants are instructed to retell the story while looking at each picture in sequence (Berman and Slobin, 1994).^3^ In this study, the wordless picture book was used to spark engagement in a social activity of storytelling. In each elicitation, I pre-identified one young adult or child to be the primary teller. I conducted the narrative elicitations in the primary teller's home. When children were primary tellers, adult caregivers and one or more of the children's siblings co-constructed the emerging narrative. The primary teller did not look through the pictures ahead of time, but crafted the narrative as they moved through the picture book.

I video-recorded all narrative elicitations, and transcribed them morpheme-by-morpheme alongside one of the child narrator's adult family members. I used the Leipzig glossing rules to annotate the data.^4^ Employing language socialization methods, I approached transcription sessions as an ethnographic practice (Schieffelin, 1990, p. 31). Adult assistants offered ongoing commentary on linguistic features as well as their beliefs about narratives and childrearing. Caregivers' interpretations and comments were included in annotations, and used as contextual ethnographic data.

With this approach to narrative elicitation, I did not attempt to use the picture book as a stimulus in a regularized experiment. Rather, I developed a method of flexible prompting, with the goal of observing how children and families responded to a novel storytelling situation. This allowed me to collect data that complemented naturalistic interaction and were appropriate for interaction analysis. Children's participation in the social settings of elicitation, as well as the form and content of their talk, serves as the primary object of analysis in this paper.

### Interaction Analysis

After collecting and transcribing the elicited narratives, I used interaction analysis to examine children's uses of evidentiality in perspective marking. Interaction analysis is derived from conversation analysis (Goodwin and Heritage, 1990) and language socialization (Duranti et al., 2014). It is an empirical method of interpreting how participants build on each other's contributions to ongoing talk. Interaction analysis takes particular interest in the sequential unfolding of talk, which provides the researcher with direct access to collaborative meaning-making (Sacks et al., 1974). That is, interaction analysis highlights the turn-by-turn processes through which participants build intersubjective alignments, using language and other semiotic resources.^5^ Interaction analysis is particularly well-suited to examining perspective marking because it reveals how participants coordinate shared and evolving knowledge states in real-time. These processes build our knowledge of language, as we respond to and identify our own and others' emotional and epistemic states. The patterns of linguistic perspective marking that interaction analysis reveals in local communicative settings intersect with broader cultural value systems (de León and García-Sánchez, 2021).

### The Linguistic Setting: Examining Perspective Marking Through Evidentiality

Evidentiality refers to a set of grammatical resources that speakers use to talk not only about *what* they know, but also *how* they know it. While speakers of all languages discuss their information sources through words, about a quarter of the world's languages include grammatical evidentiality, or morphemes that speakers *must* use to specify the knowledge source behind every utterance. Evidential morphemes can express additional meanings alongside knowledge source. This is true in Tibeto-Burman languages, where evidential morphemes also express time (tense/aspect) and subjectivity (self vs. others' perspectives). In Amdo Tibetan, evidential morphemes encode participants' positionality in relation to one another and their objects of joint attention. Specifically, Amdo evidential markers differentiate three major categories of knowledge: (1) “egophoric” evidentials articulate knowledge gained through personal involvement or experience; (2) “factual” evidentials articulate general knowledge; and (3) canonical evidentials articulate contingent knowledge (DeLancey, 2018, p. 580).^6^ Canonical evidentials can articulate either sensory perception such as witnessing (“direct evidential”), or inference (“indirect evidential”). The three categories of egophoric, factive, and canonical evidential are affixed to finite clauses as suffixes or clitics, and differ based on the phrase's tense/aspect, as well as the main verb's class.

In Amdo narratives, the use of non-stative verbs in the perfective and progressive aspects is most salient. When analyzing the elicited narratives, I therefore focused on evidentiality in these specific verb configurations. Amdo speakers discussing completed past events (the perfective aspect) must choose from amongst four different suffixes: (1) zero marking (or –*a*) (egophoric); (2) –*n*ə*.re* (factive); (3) –*t*^*h*^*a* (canonical direct); (4) and –*z*$\overset{⇁}{k}$ (canonical indirect). Speakers discussing ongoing events (the progressive aspect) must choose from amongst three different suffixes: (1) zero marking (egophoric); (2) –*n*ə*.re* (factive); (3) and –*k*ə (canonical direct). Table 2 summarizes these morphemes.

**Table 2:** Amdo perfective and progressive evidential markers.

	**Egophoric (EGO)**	**Factive (FCT)**	**Canonical Evidential: Direct (DE)**	**Canonical Evidential: Indirect (IE)**
Perfective	*∅/-a*	–*nə.re*	–*t^*h*^a*	–*z*$\overset{⇁}{k}$
Progressive	∅	–*nə.re*	–*kə*	–

The categories of egophoric, factive, canonical direct, and canonical indirect allow speakers to indicate their perspectives on sequenced events. I counted the total number of tokens of each perfective evidential marker by primary teller, to illustrate the considerable variability in children's repertoires, as opposed to adults' more regularized repertoire (Tables 3, 4). I attributed these differences to the pragmatics of the interactional setting, which led interlocutors to use evidentials to focus on distinct narrative dimensions (Ochs and Capps, 2001, p. 20). While adults' monologic narratives centered on narrated events, or those events unfolding in the world of the story, children's dialogic narratives highlighted narrative events, or the social world of storytelling.

**Table 3:** Adult's elicited monologic narratives: perfective evidential markers.

Age of Narrator	20	21	21	21	22
Tokens of EGO (∅)	0	0	0	0	0
Tokens of FCT (–*nə.re*)	0	0	2 in quoted speech	0	0
Tokens of DE (–*t^*h*^a*)	0	0	1 in quoted speech	0	0
Tokens of IE (–*z*$\overset{⇁}{k}$)	29	18	19	36	16
Total perfective clauses	29	18	22	36	16

**Table 4:** Children's elicited narratives: perfective evidential markers of primary tellers.^7^

Age	4;0	4;2	4;4	4;5	5;7	5;10	5;10	6;9	7;3
Tokens of EGO (∅)	4	1	8	12	13	2	6	11	8
Tokens of FCT (–*nə.re*)	0	8	2	11	17	15	46	28	5
Tokens of DE (–*t^*h*^a*)	7	16	3	5	1	0	0	0	1
Tokens of IE (–*z*$\overset{⇁}{k}$)	7	1	14	4	7	5	3	0	5
Total perfective clauses	18	26	27	32	38	22	55	39	19

I focused on evidentiality because this grammatical system is a context-dependent way of marking perspective. While evidentiality was originally understood as speakers' marking of “attitudes toward knowledge” (Chafe and Nichols, 1986, p. vii), later crosslinguistic studies defined evidentiality more narrowly, as the grammaticalized marking of information source (Aikhenvald and Dixon, 2014). While the question of whether the core semantics of evidential markers articulate information source, speakers' perspectives on their knowledge, or additional epistemic meanings remains a subject of lively debate amongst typologists, scholars agree that Tibetan paradigms of evidentiality include markers of subjectivity and therefore serve to mark perspective (Hill and Gawne, 2017). Regardless of researchers' stances on the universal semantics of evidentiality, studies of the pragmatics of adult narratives note that evidentiality differentiates self/other perspectives (Mushin, 2001; Tournadre and LaPolla, 2014, p. 241; DeLancey, 2018; Howard, 2018; Nuckolls, 2018). Several studies of evidentiality in adults' conversational sequences show that speakers choose evidential markers based on sensitivity to their interlocutors' perspectives (Dwyer, 2000 on Salar; Gipper, 2011 on Yurakare; Gawne, 2013 on Yolmo). In other words, speakers' uses of evidential morphemes demonstrate how they position their emotional and epistemic states in social contexts. The literature on perspective marking through evidentiality provides an empirical foundation for using interaction analysis to interpret the pragmatics of Amdo Tibetan evidentiality, and to connect these findings to the cultural values observed through ethnographic participant observation.^7^

### The Ethnographic Setting: Participant Observation as a Pathway to Narrative Elicitation

My analysis is informed by 15 months of language socialization research (2016–2018) that resulted in a corpus of over 65 h of spontaneous language data. The narrative elicitations examined in this paper complement a longitudinal research project, which compared the language socialization of four Amdo Tibetan children aged 1–4 who were growing up in rural and urban settings.^8^ All participants were native speakers of Amdo Tibetan, a language with an estimated 1.8 million speakers (Ethnologue, 2020).^9^ The participants all reported Farmer Talk (Tib. *rong skad*),^10^ the variety of Amdo Tibetan shared amongst farming communities in Qinghai, as their mother tongue. Due to significant regional variation between forms of Amdo Farmer Talk, I only enrolled participants who were living in or traced their heritage to Tsholho Tibetan Autonomous Prefecture, in Qinghai province.

Despite ongoing shift to the dominant language of Mandarin, Amdo Tibetan Farmer Talk was the primary medium for language socialization in early childhood in all focal families. Due to the continued acquisition of Amdo Farmer Talk amidst significant pressures for language shift, the grammatical details of young children's communicative practices are an important avenue of investigation to ensure cultural and linguistic survival. The broad goal of this longitudinal research was to examine how a host of economic and cultural changes were affecting young Amdo children's social and linguistic development. Amdo communities are facing urbanization on an unprecedented scale, the recent introduction of Mandarin as a lingua franca, and restrictions on traditional livelihood strategies (Yeh and Makley, 2019). In this context, Amdo parents expressed considerable anxiety about the loss of peer group play as the primary setting of young children's socialization, and noted pressure to socialize their children through mainstream schooling and structured extracurricular activities. Amdo parents expressed concern that the youngest generation's social development would suffer from a lack of strong peer relationships built in early childhood. Amdo parents also associated language shift to Mandarin with children's lack of access to close-knit peer relationships in their early socialization (Ward, 2019, p. 156–157). Due to these linked anxieties about language shift and social development, cultivating Tibetan moral values became a focal point of everyday communicative routines. Narrative was a key discourse genre through which parents addressed these concerns.

My decision to isolate Amdo's evidential system for analysis using a picture book elicitation prompt emerged from participant observation, which highlighted moral concerns in local theories of childrearing. In everyday talk to young children, adults told elaborate stories with the explicit goal of teaching them compassion (Tib. *snying rje*). Compassion is a Tibetan Buddhist value, which emphasizes avoiding harm to all living beings. Amdo adults used the value of compassion to ensure the cultural reproduction of their community, and to mark their difference from other ethnolinguistic groups (Ward, 2019, p. 191–194). Amdo adults even used narratives as a form of discipline, aiming to teach their children appropriate ways of articulating emotional states and performing sociality. In the course of participant observation, Amdo adults described children's stories as a form of positive discipline that increased loving relationships between parents and children (Ward, 2019, p. 197).

The same emphasis on compassion crosscut discourse about narrative, everyday disciplinary practices, and explicit moral socialization. In rural and urban homes, spontaneous narrative practices ranged from highly structured, involving an extended monolog to a captivated audience, to highly spontaneous and improvised. In structured narratives, a single caregiver would read a picture book or tell a monolog to multiple children. Picture books depicting traditional Amdo children's tales became readily available by the early 2000s, and are increasingly popular within China and abroad.^11^ In spontaneous narratives, adults told improvisational stories, often using animal characters, to shape children's behavior. For example, when children dug up plants or handled insects, a supervising adult often launched into a spontaneous narrative that described the feeling states of the affected creatures. These spontaneous narratives functioned as discipline by emphasizing other beings' emotional responses, rather than by issuing explicit directives (Ward, 2021).

These details of the ethnographic setting, the community's moral orientation toward compassion, and family's uses of orality and literacy are relevant to my methodology. My motivation for developing the narrative elicitation task examined in this paper arose by identifying: (1) points of particular discursive emphasis in adults' everyday talk about children's social and linguistic development, (2) salient communicative routines in children's moral socialization that focused on cultivating compassion through narrative, and (3) cultural norms surrounding literacy and orality in storytelling.

## Results

Adults and children framed narrative elicitation tasks differently. This contributed to unique patterns in the observed narrative repertoires, and highlighted how Amdo children use evidentiality in a range of functions sensitive to the social world of storytelling. As primary tellers, Amdo adults framed the narrative elicitation task as a monolog. They consistently used evidentiality to differentiate their subjective perspectives from those of the story's characters. This heightened attention to distinguishing the perspectives of self and other formulated a genre of fictional children's stories that adults described as consistent with moral socialization into compassion. Although crafted for a child audience, adults' narratives focused on the world of the story. In contrast, when children were primary tellers, the narrative elicitation task became dialogic. Collaborating children and adults used a more diverse set of evidential markers, focusing on the social world of storytelling. When children responded to adults' sequential contributions, they mirrored adults' uses of evidentiality, displaying sensitivity to their interlocutors' perspective-marking.

This section discusses three significant findings: (1) Amdo adults' monologic folktales showed patterned uses of evidentiality that relate to the community's emphasis on moral socialization; (2) In dialogic Frog Story narratives, children used evidentiality to align with their co-tellers' perspective-marking; (3) In dialogic Frog Story narratives, adults asked prompting questions to scaffold children's production of the conventionalized narrative genre.

### Adults' Perspective Marking in Elicited Monologic Narratives

When adults were positioned as primary tellers of folktales, they created monologic narratives that consistently distinguished their own narrative voice from the characters' experiences and internal states. In fact, as primary tellers, adults marked *all* past events with the indirect evidential, except when embedding characters' perspectives in reported speech (Table 3). This form of perspective marking was an affective display crafted for children's moral socialization. With this conventionalized use of indirect evidentiality, adults emphasized that they did not directly witness or experience narrated events. This narrative strategy highlighted the direct experiences and internal feeling states of the characters. By focusing on characters' direct experiences, adult narrators oriented children toward compassion without explicitly directing their behavior. In other words, adults used perspective marking to articulate a culturally valued disposition, which requires attention to the boundaries of egocentric knowledge.

In example one, Dolma Tso, a young adult narrator, retold a common folktale:

**Example 1**: Dolma Tso (age 21)^12^


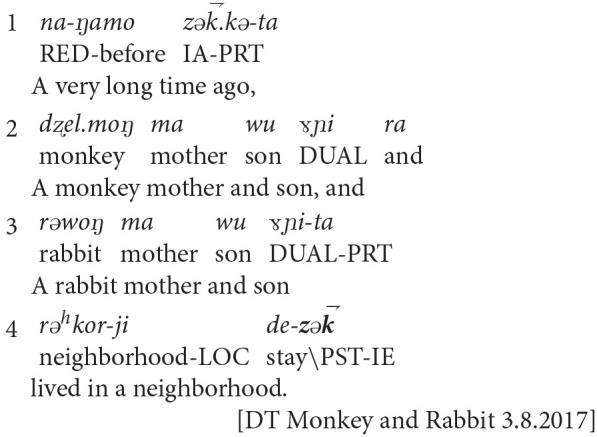


Dolma Tso used the indirect evidential to close the opening sequence (line 4). This narrative strategy depicted a fictional, temporally distant narrative setting, and backgrounded plot events to make characters' direct sensory experiences and feeling states more apparent.

When using reported speech, the same narrator articulated characters' experiences with the direct evidential. In example two, Dolma Tso told a story about a shepherdess who came across a monster while grazing her sheep on a mountain. The monster ate several of the sheep in her care and threatened to eat her, as well. The next day, the shepherdess was crying as she walked along the mountain trail, frightened but resigned to her fate of being eaten. However, she suddenly encountered a rabbit. With the help of the wily rabbit, the shepherdess was able to capture the monster and throw him off the mountain. In example two, Dolma Tso was recounting the moment when the shepherdess met the rabbit on the trail, and the rabbit asked her why she was crying:

**Example 2**: Dolma Tso (Age 21)


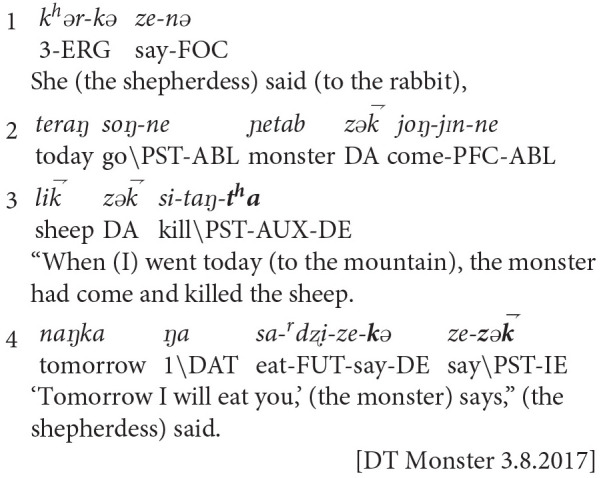


Dolma Tso contrastively used evidentials to shift amongst three perspectives: her own, that of the shepherdess, and that of the monster as reported by the shepherdess. In line 1, she introduced the reported speech event. In lines 2-3, she quoted the shepherdess' speech to rabbit, and closed the finite clause with the perfective direct evidential marker (–*t*^*h*^*a*). This use of the direct evidential is embedded in the shepherdess' speech to the rabbit, showing that the shepherdess had personally witnessed the monster killing the sheep. In line 4, the narrator doubly embedded reported speech. She voiced the shepherdess' quotation of the monster's speech with progressive direct evidential (–*k*ə). When closing the event sequence, the narrator shifted back to her own perspective. She encoded the matrix verb of speaking with the perfective indirect evidential (–*z*ə$\overset{⇁}{k}$). With this switch, the narrator transitioned from speaking as the shepherdess to speaking as herself. The narrator differentiated the character's direct experience of being verbally threatened from her own indirect knowledge of the narrative's plot. This sequenced calibration of self/other perspectives marks the boundaries of access to others' internal states and lived experiences. The narrator's consistent marking of experiential distance heightens the audience's sensitivity to characters' internal states, focusing attention on the internal story-world.

### Children's Elicited Narratives: Aligning Perspectives in Collaborative Storytelling

Children's elicited narratives were highly collaborative. Possibly because children do not tend to take on the role of sole narrator in spontaneous moral socialization, siblings and parents participated in narrative elicitation tasks by prompting the child who had been identified as the primary teller. This different social orientation to the elicitation task resulted in a focus on the narrative events in the social world of storytelling, as opposed to the story-world itself. In other words, participants more explicitly oriented toward each others' unfolding perspectives.

In their elicited narratives, children showed variable uses of evidentiality, producing the full range of evidential morphemes in perfective finite clauses with non-stative verbs (Table 4). Children produced evidential marking far more frequently than in everyday talk. However, the diversity in children's evidential configurations suggests that, even by age seven, they do not use the pattern of indirect evidential marking found in adults' monologic narratives. The variability in children's uses of evidentiality falls in line with previous experimental findings on the expected developmental trajectory for acquiring this grammatical system. Previous experimental studies suggest that, even by middle childhood, children do not fully comprehend evidential contrasts (Ozturk and Papafragou, 2015). An ethnographic reading of these elicited narratives, however, requires us to address the fact that family members did not position children as sole narrators. When a child was identified as the primary teller, adults and other children took on roles as active co-tellers. When collaborating in children's narrative elicitations, adults also used a wider range of evidentials than in their own monologic narratives. These more variable evidential configurations arose because, when families framed the elicited narratives as dialogic, adults and children moved more actively between the internal story-world and the social world of storytelling.

Interaction analysis therefore suggested that the social setting of the narrative elicitation task, as well as the participants' aged identities (Berman, 2019, p. 45–46), shaped the resulting repertoires of evidentiality. Instead of presenting a fixed perspective on narrated events and adopting a consistent emotional disposition, adults and children oriented toward each other's unfolding knowledge in the course of the narrative elicitation task. Examining the sequenced uses of evidentiality in these dialogic narratives provides insight into children's competencies in calibrating their perspectives to the social world of storytelling. Both adults and children coordinated their evidential usage with previous turns at talk, collaboratively building a shared perspective on the narrated events. Therefore, when children produced evidentiality in narrative, they were highly attuned to other participants' interactional cues.

When peers collaborated with the primary teller, the children built a shared perspective on narrated events by mirroring each other's evidential marking. They also marked the ends of event sequences by using evidentials to shift the perspective that had been established in previous turns. In example three, Sakya (aged 3;8), the primary teller, used contributions from her 3-year-old cousin (C) and her mother (M) to advance the plot sequence. Sakya had flipped the page of the Frog Story picture book, to an image where a swarm of bees rose out of a hive and chased the child protagonist and his dog. Sakya and Cousin narrated this event, before Mother turned the page to reveal that the child had escaped by climbing up a tree. Mother turned the page one more time, showing an image of the child falling from the tree.

**Example 3:** Sakya Dolma (age 3;8), Cousin (age ~3), Mother


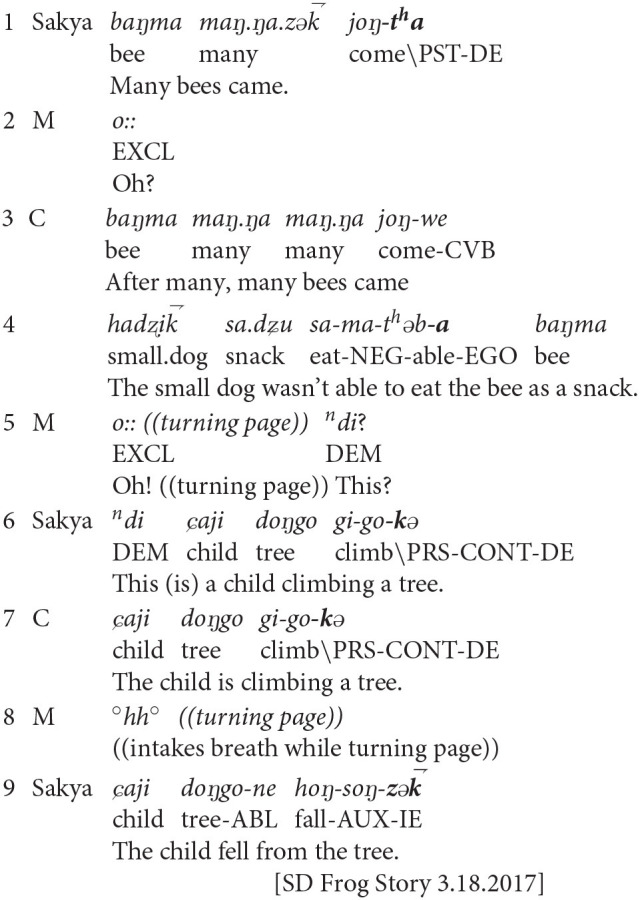


In example three, Sakya and Cousin used evidentiality to align their perspective marking across three unfolding events: the arrival of the bees, the child climbing a tree to escape the bees, and the child falling from the tree. While the children's shared perspective focused on the images, themselves, Mother provided subtle backchannel cues to prompt the children to sequence the plot.

In line 1, Sakya used the direct evidential (–*t*^*h*^*a*) to mark the bees' arrival. Sakya's use of the direct evidential emphasized her own witnessing of the picture, which positions this event in the world of storytelling rather than the world of the story. That is, Sakya privileged her visual knowledge of the event from the picture book's images, but did not advance the plot sequence. In line 2, Mother provided a backchannel cue, which may have prompted Sakya to articulate a subsequent event. In lines 3-4, Cousin explained the outcome of the bee's arrival, using the egophoric evidential (–*a*). Cousin responded to Sakya's previous utterance by suggesting her subjective interpretation of the image in the picture book. Like Sakya, Cousin positioned the bees' arrival in the immediate interactive setting by describing her personal observations of the picture book itself. In line 5, Mother turned the page to prompt the children's continued narration. In line 6, Sakya used the direct evidential, this time in the progressive aspect. In line 7, Cousin mirrored Sakya's evidential usage. In these two lines, Sakya and Cousin emphasized their shared visual access to the image of the boy climbing the tree; they emphasized their emergent knowledge of the picture. In line 8, Mother prompted the children to advance the plot by turning the page and issuing an inbreath to indicate anticipation. In line 9, Sakya responded by reporting that the child fell from the tree, now with the indirect evidential in the perfective aspect (–*z*$\overset{⇁}{k}$). Line 9 represents a shift in Sakya's perspective. Rather than emphasizing her ongoing visual access to the images, Sakya marked the sequence's closure by expressing epistemic distance, which positioned the narrated event in the world of the story. Sakya emphasized the sequence's closure following Mother's affective display of anticipation with her inbreath. With this shift, Sakya may have been responding to Mother's paralinguistic cue to advance the plot sequence.

In example three, Sakya and Cousin co-constructed an event sequence primarily by emphasizing their shared visual knowledge of the picture book. Following Mother's cues to advance the plot, Sakya shifted her perspective to resolve an event sequence. When she did so, Sakya also displaced her own subjective perspective, and positioned the child's final act of falling from the tree within the realm of the story-world.

Not all event sequences involved children's displacements of their subjective perspectives. In example four, two children sequenced events while continuing to privilege the social setting of collaborative storytelling. Lhamo (age 4;2) was the primary teller, and was looking at Frog Story's opening pages. In these images, the child protagonist is pictured leaning out of a window along with their dog, calling out for their lost pet frog. The dog's head is stuck inside the pet frog's empty jar. In the adjacent page, the dog is pictured falling out of the window. When Lhamo hesitated to move the narrated events forward, her older sister, Dolma (age 5;10), latched onto her utterances to prompt her.

**Example 4:** Lhamo (age 4;2) and sister Dolma (age 5;10)


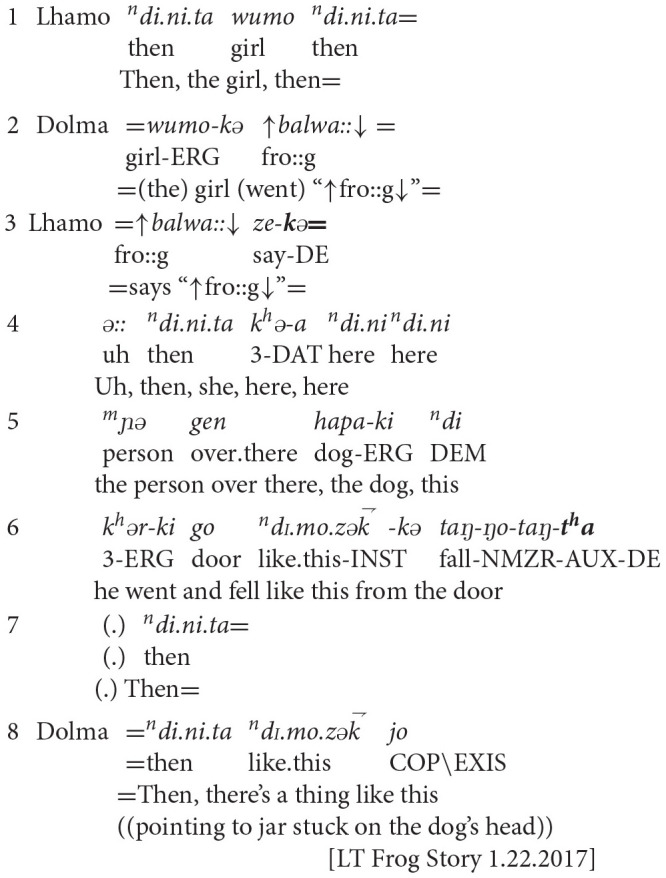


In line 1, Lhamo spoke fluently but did not advance the plot. In response, in line 2, Dolma interjected, imitating the child's act of calling out to their frog. In line 3, Lhamo latched onto Dolma's utterance, repeating Dolma's voicing of the child by reproducing her intonation. Lhamo closed the child's quoted speech with a verb of speaking, using the progressive direct evidential. This construction demonstrated Lhamo's integration of Dolma's suggestion into the plot; when she formulated the narrated event, Lhamo emphasized her witnessing of Dolma's enactment, thus focusing on the social world of storytelling. In line 4, Lhamo began to formulate the subsequent event with word searching. By line 6, Lhamo had conceptualized the event, and reported that the dog fell from the window (“door”) with the perfective direct evidential. Again, Lhamo emphasized her own sensory knowledge of the events, positioning the completed event within her domain of knowledge about her immediate social setting. In line 8, Dolma drew on a lexical item in Lhamo's previous utterance (“like this”) to provide additional information about the image.

Example four shows how Lhamo and Dolma co-constructed a narrated event sequence while remaining grounded in the storytelling world. The two children built upon each other's previous utterances to advance the plot sequence. They portrayed the unfolding events through their visual access to the picture book's images, and their aural access to the sonic form of each other's contributions.

### Interactional Scaffolding: Children's Socialization to a Narrative Discourse Genre

In the course of narrative elicitation tasks, adult's contributions offered a form of scaffolding, where they prompted children to advance a plot sequence situated within the story-world. Adults and children alike drew on the pragmatic properties of evidentiality to access the story-world. Amdo Tibetan evidentials feature an “anticipation rule” (Tournadre and LaPolla, 2014, p. 245) meaning that, when speculating or asking about another's experience, speakers use the evidential marking they expect their addressee to use in response. In question-answer sequences, this anticipation rule is particularly pronounced, because the responsibility for articulating knowledge source shifts from the speaker to the addressee. With their questioning prompts, adults mediated children's displacement of events from the world of storytelling into story-world.

In example five, Tashi (age 4;4) constructed her Frog Story with extensive prompting from her Mother. When formulating her questions, Mother managed her expectations about the conventionalized framing of narrated events in light of Tashi's contributions. Tashi and Mother moved between the world of storytelling, where they co-constructed visual knowledge of pictures, and the story-world, where they displaced an event sequence.

**Example 5:** Tashi (age 4;4) and Mother (M)


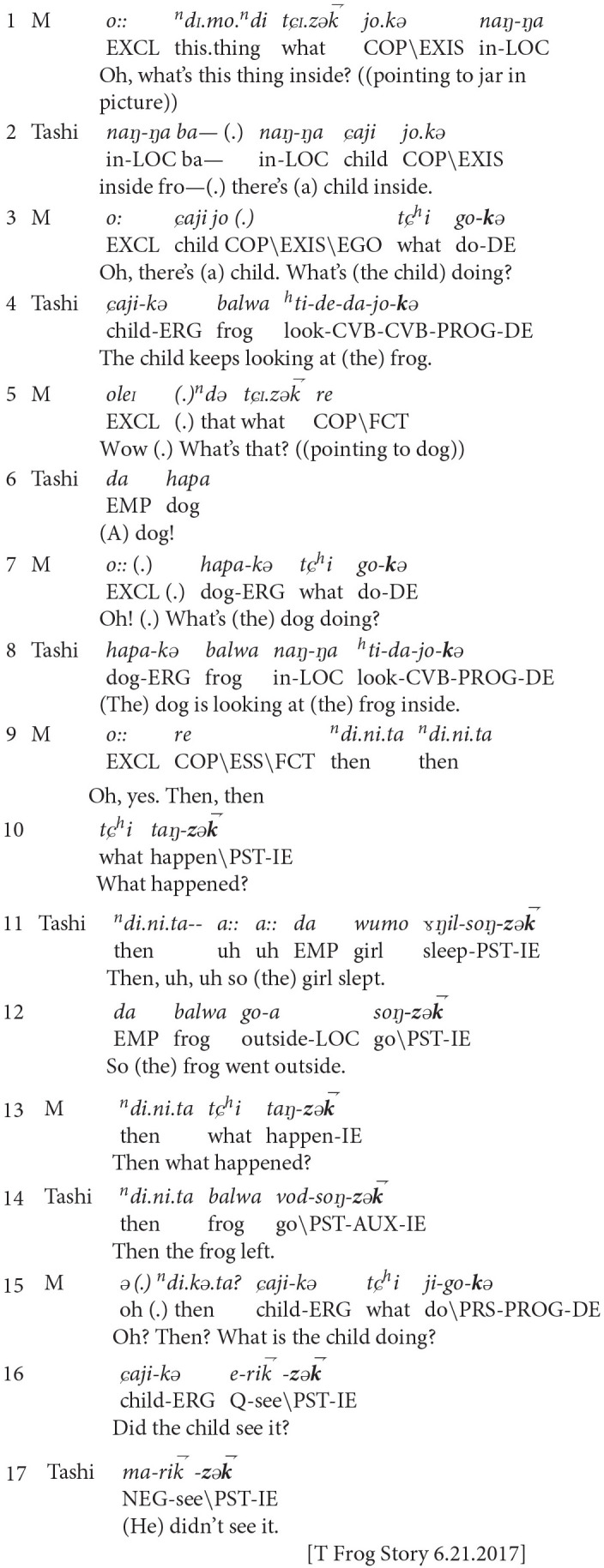


In this excerpt, Mother and Tashi move between the social world of storytelling and the narrative-internal story-world. In line 1, Mother prompted Tashi to describe the picture by asking what is inside the jar. In line 2, Tashi responded, noting the presence of the child. In line 3, Mother acknowledged Tashi's statement through repetition. She then asked about the child's ongoing activity, using the progressive direct evidential. In line 4, Tashi responded by describing the child's action of looking at the frog. Tashi used the same evidential construction as in Mother's question. In this question-answer exchange, Tashi and Mother highlight their shared visual knowledge of the pictures, remaining focused on the social activity of co-telling. This orientation continued as they described the ongoing narrated events using the progressive aspect in lines 5–8.

In lines 9–10, Mother shifted their joint orientation. She used the indirect evidential in the perfective aspect to ask Tashi to describe the next event. This prompted Tashi, in lines 11–12, to displace the event sequence into the story-world. Tashi again used the same evidential configuration as Mother. At this point, Mother and Tashi have shifted from describing each image in detail, to conveying a coherent event sequence. Mother's prompting questions led Tashi to use indirect evidential marking, characteristic of the conventionalized Amdo Tibetan narrative discourse genre, to advance the narrated events.

After Tashi and Mother established the temporal frame of the event sequence in lines 9–12, they continued to remain within the story-world by marking perfective clauses with the indirect evidential. In her prompting questions, Mother used the perfective indirect evidential to scaffold Tashi's advancement of the plot sequence. When Tashi omitted significant details that were depicted in the picture book, Mother used direct evidentials to shift their orientation back to picture description in the storytelling world. In each of Tashi's responses to Mother, she mirrored Mother's evidential marking.

In line 13, Mother framed the narrated events in the perfective aspect with the indirect evidential. In line 14, Tashi mirrored Mother's evidential configuration, explaining that the frog left. In line 15, Mother asked a descriptive question about the picture with the progressive direct evidential. In line 16, she followed with a question about the upcoming plot event, using the indirect evidential. In line 17, Tashi responded by reframing her mother's question into a negative assertion with the same evidential marking.

Example 5 demonstrates how the interlocutors' ongoing mutual orientation established distinctive frames that moved between the narrative events in the storytelling world and narrated events in the story-world. Mother's prompting questions shifted from reporting on immediate sensory input in the social situation of telling to describing a displaced, sequenced narrative. Mother's shifts in perspective responded to Tashi's ongoing contributions, as well as the cultural expectations of a narrative discourse genre. Mother therefore scaffolded Tashi's performance of a conventionalized narrative through her prompting questions. In so doing, she positioned Tashi as a novice narrator. Mother took on an active role in shaping the trajectory of Tashi's story-world, as well as the interactive setting of the storytelling world.

Collectively, examples 3–5 suggest that children tended to use evidentiality to prioritize their immediate experiences in the social situation of the story-telling world. Co-present adults' prompted children to advance a narrated plot sequence by displacing events in a past time and articulating their indirect knowledge of the narrated events. That is, adults attempted to guide children's narratives toward a conventionalized genre, which marks epistemic distance from the world of storytelling and focuses on characters' internal experiences. As Takada and Kawashima (2019, p. 216) found in a study of Japanese families' collaborative picture book reading, caregivers tended to shape toddlers' turns “according to the socioculturally structured script of the story.” More specifically, caregivers guided toddlers to sustain plot sequences by associating events in the story-world with the immediate interactive setting. In Amdo Tibetan elicited narratives, adults similarly guided children toward the cultural expectations surrounding monologic narratives. Adults used prompting questions to encourage children to advance plot sequences instead of describing each picture, and to mark epistemic distance from narrated events in the story-world. These prompting questions suggest that adults were positioning children as novices. However, children's ability to respond to these cues hinged on their communicative competence in the pragmatics of evidentiality. Even though Amdo children did not tend to produce grammatical evidentiality in their spontaneous talk, their contingent responses as narrative co-tellers demonstrated their understanding of how to use this grammatical system to calibrate perspective marking. With their understanding of how evidentials mark shifts in knowledge between speaker and recipient, children were able to competently respond to adults' prompting questions, using evidentials to displace events into the story-world.

## Discussion

In the face of rapid social and economic change, Amdo Tibetan communities are grappling with anxieties about cultural and linguistic survival. These anxieties contribute to Amdo Tibetan caregivers' heightened focus on the everyday moral socialization of their young children through narrative. In Amdo Tibetan families, narratives for children aim to cultivate strong social bonds, along with Buddhist senses of compassion (Tib. *snying rje*) for all living beings. As a discourse genre, adults' narratives for children show preferences surrounding the use of certain grammatical features, especially evidentiality. Building on previous language socialization literature, which has demonstrated how patterned uses of grammatical features contribute to the socialization of emotion, this paper examined links between evidentiality and the verbal repertoires meant to cultivate a compassionate disposition. Despite the prominence of narrative and evidentiality in language input to children, Amdo Tibetan children's spontaneous language data included very few tokens of evidential markers. Looking exclusively at adults' language production to understand the links among narrative, patterned uses of grammatical perspective marking, and cultural values would present a partial understanding of how children come to acquire this distinctive communicative style. By putting diverse data sets into conversation with one another through a narrative elicitation task, this paper has shown how we can locate patterns in children's experiences of language across complementary settings of data collection, in a community where children's acquisition of their mother tongues is a point of particular concern. Using narrative elicitation as a tool in ethnography can reveal connections between the grammatical details of communicative routines, a community's cultural values, and how children are positioned as subjects or agents of socialization in certain discourse genres.

Interaction analysis of Amdo adults' elicited narratives showed cultural preferences surrounding perspective marking using evidentiality. Examples one and two demonstrated that, in monologic narratives, adult speakers clearly demarcated their indirect perspectives from the characters' direct sensory and experiential knowledge of the unfolding narrated events. Scholars examining adults' monologic narratives in diverse languages including Pastaza Quichua (Nuckolls, 2018), highland Quechua (Howard, 2018), and Central Tibetan (Ward, 2016; DeLancey, 2018) have found that strategies of perspective marking interface with cultural values surrounding morality. In the ethnographic context of Amdo Tibetan communities, this strategy of perspective marking aligns with caregivers' concerns over children's moral socialization. In narrative, adults use perspective marking to heighten their child audience's sensitivity to the internal states of others. This grammatical emphasis on others' sensory experiences provides a culturally-valued form of discipline, which teaches children to orient toward others' feeling states and recognize the epistemic boundaries of their personal experiences.

When approaching the narrative elicitation task, community members positioned adults as socializing agents. Adults crafted monologic narratives for an imagined child audience. In contrast, when the researcher positioned children as primary tellers, parents and co-present children collaborated as tellers of dialogic narratives. Interaction analysis of these elicited dialogic narratives found that: (1) children collaboratively coordinated a shared perspective by using evidentiality to focus attention on the social world of storytelling; and (2) adults' contributions to the narrative elicitation task provided a form of scaffolding that prompted children to advance plot sequences and produce the patterns of perspective marking that characterize monologic narratives. Specifically, in examples three and four, young children aligned their perspectives by co-constructing narrative event sequences focused in their immediate social interaction. Their uses of direct evidentiality responded to ongoing sensory input, including their visual access to the picture book and their co-present interlocutors. Young children responded to cues to displace events into the realm of the story, by shifting their uses of evidentiality to close or advance plot sequences. In example five, a mother prompted her daughter to move between the world of storytelling and the world of the story, by advancing the narrated events through a series of questions and answers. By mirroring her mother's use of the indirect evidential, the daughter approached grammatical conventions for depicting narrated events from within the story-world.

Previous crosslinguistic studies using Frog Story have found a tendency for younger children to interpret the elicitation task as picture description, but for people aged nine and above (including adults) to construct globally cohesive storylines (Berman and Slobin, 1994, p. 69). However, drawing on Berman's (2019) attention to the role that ideologies of age play in shaping children's verbal repertoires, we can call into question whether the differences in Amdo children's and adults' narrative repertoires may, in fact, result from aged identities rather than chronological age. Because children were not positioned as socializing agents with the authority to tell monologic narratives, their repertoires of evidentiality responded to the social setting of storytelling more clearly than those of adults'.

The dialogic narratives demonstrated Amdo children's sensitivity to the pragmatics of evidentiality in situated perspective marking. Children aligned their evidential configurations with their interlocutors' previous turns. These findings provide a window into children's communicative competence with a syntactic system that requires reflection on others' knowledge and appropriate displays of internal states. Previous crosslinguistic studies of evidentiality in interaction (Dwyer, 2000; Gipper, 2011; Gawne, 2013; San Roque, 2015) found that adult speakers calibrate evidential marking to the expectations of their conversational partners. Research on children's uses of these syntactically embedded and polysemous grammatical systems can help demonstrate how children respond to cultural norms surrounding sociality, even when their uses of these systems are restricted in spontaneous talk. For example, San Roque and Schieffelin (2018) examined uses of the semantically-related system of egophoric marking in Kaluli (Papua New Guinea) question-answer pairs. Their findings suggest that situated uses of egophoric marking may help socialize children into cultural beliefs surrounding epistemic authority, or who can say what about their own or others' experiences. Interaction analysis of Amdo children's narrative elicitation tasks shows that children coordinate their attention to sensory input and others' interactional cues when learning how to grammatically encode epistemic and emotional states.

Adults' and children's framings of the narrative elicitation task also provided evidence of how the cultural construction of aged subject positions may influence verbal repertoires. Because elicitation tasks result in less ‘natural’ speech (Klamer and Moro, 2020), an ethnographic attention to this methodology can provide insight into the broader cultural norms and communicative ideologies that influence who does or can produce a given repertoire. For example, de León (2009) found that, while Tzotzil Mayan children's spontaneous narratives used a rich repertoire of evidentiality characteristic of their community's narrative discourse genre, their elicited Frog Stories tended to lack evidential marking. Elements of the narrators' backgrounds, including years of schooling and the use of Spanish, were correlated with different repertoires of evidentiality in elicited Frog Stories (ibid, p. 187). These stark distinctions between spontaneous and elicited Tzotzil narratives demonstrate that a community's framing of elicitation tasks influences grammatical patterns in the resulting stories. By similarly examining Amdo Tibetan families' framings of a narrative elicitation task, this study revealed the cultural expectation that adults' monologic narratives should serve as a socializing tool for children. Children, in contrast, produced dialogic narratives that revealed their competencies in coordinating perspective marking through evidentiality, a skillset which was under-represented in spontaneous talk.

Approaching Frog Story elicitations as ethnography provides new possibilities for a method traditionally used in experimentation or larger-scale, cross-sectional analyses. In the discipline of Psychology, studies of children's language development have used narrative elicitation as a tool for building databases that facilitate comparison across age ranges, language communities, and social settings (Berman and Slobin, 1994; Stromqvist and Verhoeven, 2004). Picture book elicitation, in particular, has been used extensively in the crosslinguistic and age-graded study of language acquisition because it presents a consistent discourse activity based on a stable stimulus (Slobin, 1985; Bamberg, 1987, p. 21). Crosslinguistic research using traditional narrative elicitation methods has extended the study of children's cognitive and linguistic development to their social and cultural knowledge, addressing children's communicative competence in the grammatical particularities of their native language(s). When analyzing regularized, monologic narratives, however, children's social and cultural experiences of language must be documented outside of the narrative elicitation task, itself. With a basis of ethnographic knowledge, researchers using this approach have independently associated narrative style with cultural values (Wilkins, 1997; Bavin, 2004).

Approaching narrative elicitations as culturally situated activities is a method particularly well-suited to examining early childhood socialization in under-documented languages threatened by language shift. In minoritized languages communities, anxieties about language loss often intersect with child-rearing practices that emphasize moral socialization through language. At the same time, language ideologies that explicitly devalue specific features or codes (Hornberger, 2008), or position these in opposition to children's identities (Ochs, 1986; Meek, 2008; Berman, 2019) prevent children from demonstrating their full communicative competencies. These ideological facets of development intersect with children's changing grammatical knowledge. Features embedded in a language's syntax, such as Amdo Tibetan evidentiality, may *never* be fully represented in a child's spontaneous language use if language shift occurs rapidly in early childhood. In this context, elicitation tasks provide novel communicative settings that can reveal children's competencies in grammatical systems that are not represented in spontaneous data. Amdo Tibetan narrative elicitation tasks presented a possible pathway toward language continuity rather than shift, by demonstrating children's ability to use interactional cues to coordinate perspective marking with evidentiality. As the current generation of Amdo Tibetan children ages, whether or not they will follow this pathway toward reproducing a culturally valued communicative style, in light of considerable pressure to adopt the dominant language of Mandarin, remains an open question.

Expanded uses of narrative elicitation in language socialization research could provide further insight into possible strategies for language preservation. In the case of Amdo Tibetan, continued research could probe the boundaries of monologic narratives, as well as the extent to which this community's repertoires of evidentiality are affected by aged identities. For example, future narrative elicitation tasks could explicitly position children as sole tellers of monologic narratives, and prompt children to tell narratives to different audiences. Combined with ethnographic attention to spontaneous narratives, these methods could reveal potentialities to nourish the production of a wider range of grammatical systems, earlier in children's development. In communities experiencing language shift, it is only by expanding children's grammatical repertoires and domains of language use that we can work toward linguistic survival.

## Data Availability Statement

The original contributions presented in the study are included in the article/supplementary material, further inquiries can be directed to the corresponding author/s.

## Ethics Statement

The studies involving human participants were reviewed and approved by New York University Institutional Review Board. Written informed consent to participate in this study was provided by the participants' legal guardian/next of kin.

## Author Contributions

The author confirms being the sole contributor of this work and has approved it for publication.

## Conflict of Interest

The author declares that the research was conducted in the absence of any commercial or financial relationships that could be construed as a potential conflict of interest.

## Footnotes

^1^All names are pseudonyms. Children's ages are given in years; months. Lhamo and Dolma participated in the narrative elicitation task more than once, to allow me to examine changes in their individual language repertoires over time for a separate project.

^2^A comparison with spontaneous narratives is beyond the scope of this article. See Ward (2021) for an examination of spontaneous interactions involving narrative as moral socialization. In these interactions, children responded to adults' narratives. Specifically, a 6-year-old child marked past sequences with direct evidentials, while younger children used directives and formulaic expressions without evidentials.

^3^Larger-scale crosslinguistic projects have used different media for elicitation tasks, including short films (Chafe, 1980) and in-person and pre-recorded narratives (Mushin, 2001).

^4^The Leipzig Glossing Rules: Conventions for interlinear morpheme-by-morpheme glosses. Available online at: https://www.eva.mpg.de/lingua/pdf/Glossing-Rules.pdf (accessed December 14, 2020).

^5^Although recent language socialization research uses interaction analysis to look at the coordination of language with other embodied communicative resources, such as touch and gaze (Goodwin and Cekaite, 2018), this paper retains a focus on a specific grammatical system. This focus is relevant to methodological issues in ethnographic research with child speakers of under-documented languages whose grammatical systems are vulnerable to endangerment. As Evans et al. (2018) argue, despite the inseparable links between language and other semiotic systems, “our understanding of the full panoply of grammatical means used across languages for intersubjective coordination remains basic” (112).

^6^There is currently no consensus on the correct terminology for these categories (for alternatives see Hein, 2001; Zeisler, 2004; Tournadre and LaPolla, 2014). I chose to follow the recent terminology of DeLancey (2018). I use the term “canonical evidentiality” to refer to Amdo suffixes that are united in a single paradigm with factive and egophoric markers, but that encode semantics of knowledge source common to the evidential systems frequently attested across languages. While currently documented Tibetan languages share this three-way evidential distinction between egophoric, factive, and canonical, the specific evidential markers are not cognates. Also, the canonical evidential category is more or less elaborated in different tense/aspect constructions across Tibetan languages.

^7^Because Sonam (age 2;8) produced very few perfective finite clauses, he is excluded from the token analysis represented in Table 3.

^8^Due to the longitudinal and holistic design of language socialization research, a small sample size of four to six focal children is standard practice (Garrett, 2008, p. 192–193). I sampled everyday talk with each child in their family home, for ~2–4 h every three months. Data collection involved both audio recordings with contextual fieldnotes, and video recordings. Decisions about whether to use audio or video were motivated by participants' concerns about confidentiality, and my concerns about the sensitive nature of conducting long-term fieldwork with a minoritized community in western China. Focal participants were recruited through my existing social connections in Xining city, the provincial capital of Qinghai province. The focal children were chosen based on their parents' mother tongue (Amdo Farmer Talk) and region of origin (Tsholho Tibetan Autonomous Prefecture).

^9^Amdo Tibetan is part of a “sprachbund,” a region where genetically unrelated languages show significant structural adaptation to one another (Janhunen, 2012). With increasing pressures to assimilate to an expanding market economy, many Amdo Tibetan communities are experiencing language shift to the state's official language of Mandarin. Despite these pressures, Amdo Tibetan is considered “stable” because it remains the primary medium for socialization in young children's family interactions [Ethnologue, 2020. “Tibetan, Amdo.” Available online at: https://www.ethnologue.com/language/adx (accessed October 6, 2020)].

^10^Tibetan words with literary equivalents are depicted using the Wylie (1959) transliteration system. Due to the phonological and grammatical differences between Amdo Farmer Talk and literary Tibetan, narratives are rendered in the International Phonetic Alphabet.

^11^Children's books are published through multiple Chinese “minority nationality presses” in major cities. Several non-profit organizations aim to expand access to children's books in written literary Tibetan, and partner with Chinese minority nationality presses to disseminate the books, including Tibetan Arts and Literature Initiative (TALI, https://talitibet.org) and the Trace Foundation (http://www.trace.org/).

^12^Examples are transcribed in the International Phonetic Alphabet. Interlinear transcription identifies morphemes using the Leipzig glossing rules (https://www.eva.mpg.de/lingua/pdf/Glossing-Rules.pdf). Evidential morphemes are bolded in the transcripts. Additional paralinguistic features are marked following the conventions of conversation analysis (Sacks et al., 1974).
